# Evaluation of intensified provider initiated testing and counselling program in Harare City, 2017–2018

**DOI:** 10.1186/s12889-021-10485-6

**Published:** 2021-03-02

**Authors:** Edias Mandere, More Mungati, Gloria Gonese, Notion Gombe, Tsitsi Juru, Simbarashe Chiwanda, Emmanuel Govha, Gerald Shambira, Mufuta Tshimanga

**Affiliations:** 1 Department of Community Medicine, University of Zimbabwe, Harare, Zimbabwe; 2 Elizabeth Glaser Paediatric AIDS Foundation, Maseru, Lesotho; 3 International Training & Education Centre for Health, Harare, Zimbabwe; 4 African Field Epidemiology Network, Kampala, Uganda

**Keywords:** HIV, Eligible clients, Provider initiated testing and counselling

## Abstract

**Background:**

Knowledge of HIV status remains a challenge despite implementation of various testing strategies including provider-initiated HIV testing (PITC). Harare City intensified provider-initiated HIV testing by targeting testing all eligible clients visiting facilities to achieve the UNAIDS first 95. This study aimed at evaluating the intervention to improve its effectiveness and inform programming decisions for universal access to HIV testing.

**Methods:**

A descriptive cross-sectional study was conducted in Harare from April to June 2019. Evaluation of the intervention was conducted using the logic model approach to assess the inputs, processes and outputs. Health workers were interviewed using an interviewer administered questionnaire. Exit interviews were conducted for eligible clients > 18 years who refused to be tested. A checklist was used to assess the inputs used and a desk review of HIV screening and testing records was done.

**Results:**

A total of (n-45) health care workers and (*n* = 70) clients were interviewed with a response rate of (92%) and (84%) respectively. The median age for clients was 31(Q1 = 24: Q3 = 38) and median years in service for health workers was 2 (Q_1_ = 1;Q_3_ = 26). Of the 133,899 clients who were eligible for testing after screening, 98,587 (74%) accepted the test leaving a gap of 35,312 (26%). However, 21/45 (47%) of health workers indicated high workload in the morning as the major reason for the leakage. In addition, 25/70 (36%) of the clients indicated long waiting time as the reason for opting out of HIV testing.

**Conclusion and recommendation:**

HIV testing coverage for eligible clients was not optimal, 26% opted out. We recommend strengthening of health facility systems such as review of patient flow, re-allocation of staff during busy HIV testing time and scaling up the use of HIV self-test kits for clients concerned with waiting time to improve HIV testing coverage.

**Supplementary Information:**

The online version contains supplementary material available at 10.1186/s12889-021-10485-6.

## Background

The World Health Organization (WHO) and the Joint United Nations Programme on HIV/AIDS (UNAIDS) recommended universal access to knowledge of HIV status under the following 5 C’s consent, confidentiality, counselling, correct results and connections [1]. Provider Initiated Testing and Counselling (PITC) opt- out strategy was recommended by WHO in 2007 for high HIV burden countries (mostly in the Sub-Saharan Africa region) to scale up access to HIV testing [2]. Despite the adoption of the recommendation about 30% of people living with HIV globally were still unaware of their HIV status by the end of 2016 [1].

By the end of 2017, an estimated 9.4 million (25%) of people living with HIV were still unaware of their status [3]. Regionally, about 24% of people living with HIV in Southern Africa were unaware of their HIV status by the end of 2017 [4]. In Zimbabwe, the Population-Based HIV Impact Assessment Survey (ZIMPHIA) 2016, estimated that 1, 3 million people were living with HIV while 74% knew their HIV status against a target of 90% giving a national gap of 16% people yet to be identified [5]. Staveteig et al., 2017 reported a 69% HIV testing coverage calculated from 16 African countries, which leaves a significant number of eligible people not tested [6].

The Zimbabwe National Guidelines on HIV Testing and Counselling clearly states that, “PITC services should be provided to all adults, adolescents and children attending all health facilities as the recommended standard of care” [7]. In response, Harare City health department intensified Provider Initiated HIV Testing (PITC) with support from partners to reduce leakages from HIV testing among eligible clients. As part of this intervention, Harare city health department was provided with additional human resources, data collection tools, tents to increase testing space, tables/chairs, and capacity building of clinic staff. The intensified PITC intervention involved screening all clients visiting the facility individually for HIV testing eligibility. Clients who are eligible are offered HIV testing and if they agree, the test is performed. The availability of additional staff was meant to ensure that all clients who need the services are assisted on time. Implementing facilities were provided with strategic information tools for data capturing, analysis, and reporting. By intensifying PITC eligible people visiting health facilities are tested for HIV and enrolled into care.

Despite implementing this intensified PITC model in Zimbabwe HIV testing coverage remains below 100% among eligible clients visiting clinics. According to a PITC study conducted in Hurungwe, Zimbabwe by Musarandega et al., 2018, 77.7% of children who were eligible for HIV testing were missed [8]. In Harare 35% of clients screened at OPD were eligible for HIV testing, but only 61% of those eligible opted in for HIV testing. It is against this background that we evaluated the intensified PITC in Harare and came up with recommendations that are aimed at improving the service.

## Methods

### Study design

**Fig. 1:**
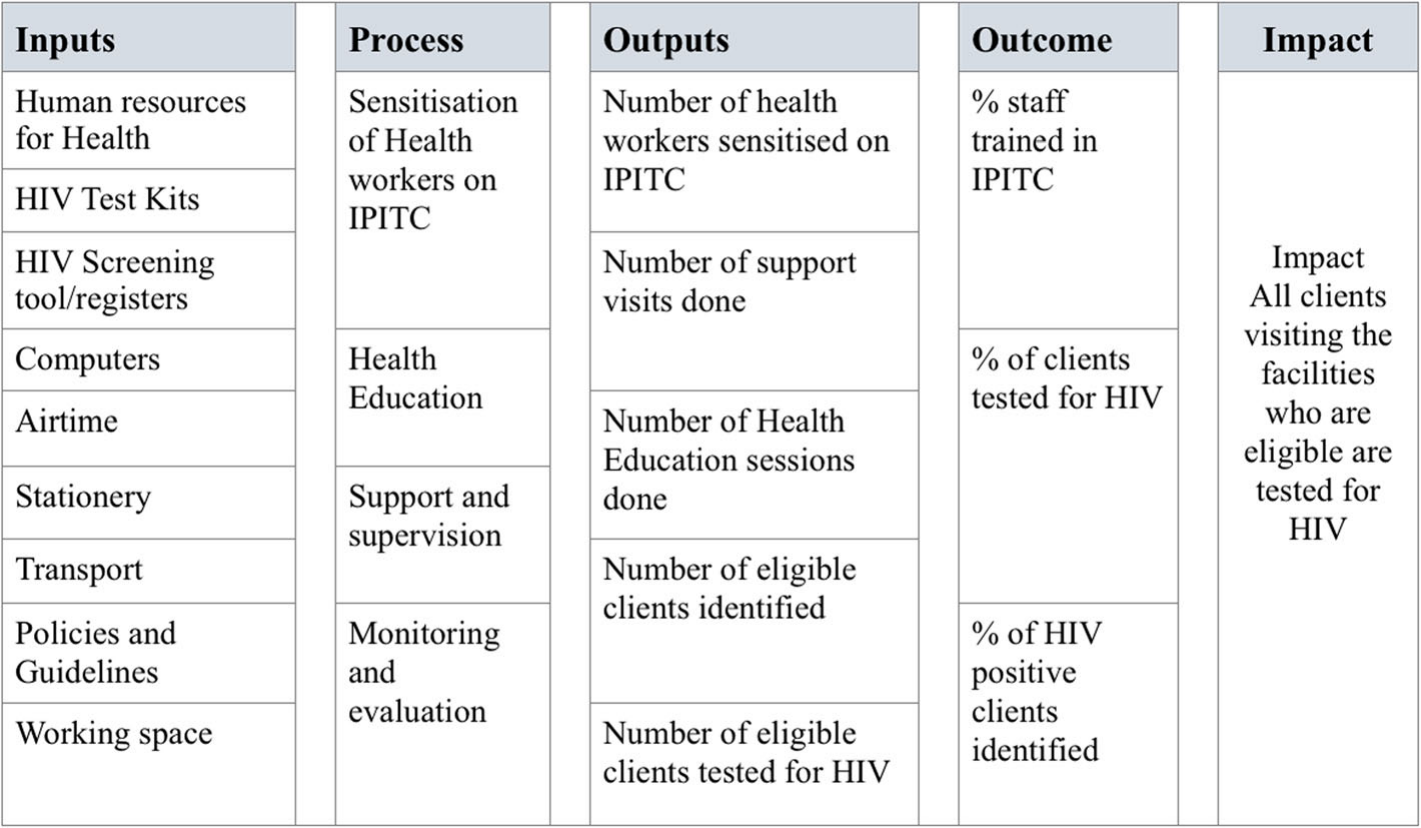
Logical model for intensified PITC Intervention, Harare City 2017–2018

We used a descriptive cross-sectional study design and conducted a process evaluation using the logic model approach. The logic model approach was preferred because it clearly depict the relationship between the problem, resources allocated and the interventions implemented to achieve the desired outcome (Fig. 1) [9]. This evaluation assessed the inputs, processes and outputs used to implement the intensified PITC intervention in Harare City .

### Study setting

The study was conducted at clinics managed by the Harare City Health Services Department. City of Harare is the capital city of Zimbabwe resident to 1,598,830 million people located in in 135 suburbs with 534,106 thousand private households [10]. A total of 42 public health facilities comprising of 12 polyclinics, 6 family health services clinics, 7 primary care clinics, 15 satellite clinics, and 2 infectious disease hospitals provide health care to residents and no-residents of Harare [11]. At each facility there are Nurses, Nursing Assistants, and Counsellors who provide HIV testing services to clients. Clients testing HIV-positive are then linked to ART and followed-up.

### Sampling and participants

Fifteen Health facilities out of the 42 in City of Harare were selected for the study. The 42 facilities were allocated unique numbers and Microsoft excel was used to randomly select the first 13 facilities. Additional 2 infectious disease hospitals were purposively selected and included in the study because of their high number of clients. Health workers found on duty were purposively recruited while Nurse Managers at participating health facilities were interviewed as key informants. Clients who opted out of HIV testing were identified using convenience sampling and interviewed on exit from the facility.

### Data collection and analysis

A structured interviewer administered questionnaire specifically designed for this study was used to determine the reasons for failing to meet the testing target by health workers (Supplementary file 1). A checklist was used to assess availability and adequacy of inputs used to implement the project. In this study adequacy was defined as an average of above (95%) achievement in terms of availability of inputs. The checklist assessed human resources, consumables, policies & guidelines, monitoring & evaluation tools, communication, and HIV testing space. A desk review of the reports and registers was conducted to evaluate the processes and outputs of the IPITC intervention. The review of records provided Information such as number of clients screened/eligible for HIV testing, number of clients accepted and tested for HIV and number of clients tested HIV positive (positivity rate). Exit interviews were conducted for eligible clients who opted out of HIV testing, to determine the reasons for refusing testing. The researcher asked the following questions for health workers and clients, respectively. What are some of the main challenges that you experienced for not meeting your HIV testing target? and What are your reasons for opting out of HIV testing?

Quantitative data was analysed using Epi-info version 7 to calculate frequencies, means and calculation of proportions. Qualitative data was sorted and analyzed manually by coding respondent’s views into similar thematic areas. We assigned labels in the form of words or phrases to represent recurring themes in participants responses. Summaries of this data was presented as frequencies and proportions of responses.

### Permissions and ethical considerations

Permission to conduct the study was obtained from the University of Zimbabwe, Department of Community Medicine, and the Health Director City of Harare. Written consent was obtained from study participants before the interviews were conducted. Privacy and confidentiality were maintained throughout the study, participants were asked if they agree to be enrolled in the study and interviews were conducted in a closed room at the health facilities. Each interview took less than 15 min to complete. Anonymity was guaranteed by not writing participants names on the questionnaires and all questionnaires were kept locked by the investigator.

## Results

A total of (*n* = 45) health workers and (*n* = 70) clients were interviewed with a response rate of (92%) and (84%) respectively. Median age for clients was 31(Q1 = 24: Q3 = 38) and the median years in service for health workers was 2 (Q_1_ = 1;Q_3_ = 26). Majority of the participants were females constituting 49 (70%) and males 21 (30%) respectively. More than half 50 (71%) of the participants attained secondary level of education. Fifty-four percent (*n* = 70) of the participants were married and half of them 35 (50%) were not employed. Twenty eight percent (*n* = 70) were earning a monthly income of between 100 and 500 Zimbabwean dollar (Table 1).

**Table 1:** Demographic characteristics of clients interviewed, Harare City 2017–2018

Variable	Frequency *n* = 70	(%)
Sex		
Male	21	30
Female	49	70
Age		
18–24 Years	18	26
24–49 Years	49	70
< 50 Years	3	4
Median age years: 31	31(Q1 = 24: Q3 = 38)	
Highest level of education		
Primary	12	18
Secondary	50	71
Tertiary	8	11
Marital status		
Single	20	28
Married	38	54
Divorced	8	11
Widowed	4	5
Employment status		
Formally employed	10	14
Informal employment	25	36
Not employed	35	50
*Monthly income (ZW$)		
< $100	26	37
100–500	28	40
> 500	16	23

*ZW$ Zimbabwe dollar

The inputs used to implement the intervention were adequate (96.8%), adequacy was defined as an average of above (95%) achievement in terms of availability of inputs. Only 1/15 (10%) facilities had a stock out of determine test kits once during the 1 year. Three out of fifteen (20%) of the facilities had their phone not working. Two out of fifteen (14%) of the facilities reported inadequate working space for testing clients especially during peak hours in the morning (Table 2).

**Table 2:** Inputs used for implementation of intensified PITC Intervention, Harare City 2017–2018

Inputs	Target	Achievement	% achievement
Human resources			
OI Nurses	30	30	100
Primary care counsellors	60	60	100
Data entry clerks	15	15	100
Consumables			
HIV test kits (Availability at facility)	15(facilities)	14	90
Gloves (Availability at facility)	15(facilities)	15	100
M & Tools			
Registers	15(facilities)	15	100
Computers	15(facilities)	15	100
Policies and guidelines			
HIV testing guidelines	15(facilities)	15	100
HIV testing algorithm	15(facilities)	15	100
Eligibility criteria SOP	15(facilities)	15	100
Communication			
Landline working	15(facilities)	12	80
Cell phones	15(facilities)	15	100
Airtime	15(facilities)	15	100
*Adequate working space			
Rooms/Tents	15(facilities)	13	86

* Adequacy of inputs is defined as an average of above 95% achievement in terms of availability of inputs

The target for processes used to implement the intervention were fairly achieved, however there were no Information Education and Communication (IEC) materials for distribution to clients to inform them about the services (Table 3).

**Table 3:** Processes used to implement intensified PITC Intervention Harare City, 2017–2018

Variable	Target	Achievement	% achievement
Training of Health Workers	1(session)	1	100
Health Education	15(Facilities)	15	100
Distribution of IEC material	15(Facilities)	0	0
Screening of eligible clients	15(Facilities)	15	100
HIV Testing (Quality Assurance)	15(Facilities)	15	100
Support and Supervision	15(Facilities)	15	100
Documentation and reporting	15(Facilities)	15	100

On outputs, the study showed that, 44/45 (98%) of health workers were trained to implement the PITC intervention at its inception in 2017. New staff who joined Harare City were trained on the job, however there were no refresher trainings for the initial group. Desk review results and program data showed that 133,899 clients were eligible for testing after screening but only 98,587 (74%) of then opted in for testing leaving a gap of 35,312(26%). The positivity rate for those tested was 7123 (7%).(Table 4).

**Table 4:** Outputs of the intensified PITC intervention, Harare City 2017–2018

Variable	Target	Achievements	%Achievements
Health care workers trained on IPITC	45	44	98
Support &Supervision visits conducted	15(facilities)	15	100
Health Education Talks Conducted daily	15(facilities)	15	100
Number eligible tested for HIV.	133,899	98,587	74
Number tested HIV Positive	6% yield	7123	7 yield

Thirty six percent (*n* = 70) of the respondents who were eligible indicated that long waiting time was the major reason for opting out of HIV testing. The other reasons cited were fear of a positive result 15 (21%) and perceived low risk of contracting HIV was cited by 15 (21%) of the respondents (Table 5). A quote from one of the participants, *“I am eligible for testing but could not wait for the test because the queue is too long, I will come back sometime”* Health workers interviewed indicated high workload 21 (47%) especially in the morning, shortage of staff 14 (31%) and poor patient flow 9 (20%) as major reasons for not meeting the target (Table 6). One of the nurses said, “*The demand for testing is usually high in the morning after screening, we need additional testers to ease the workload*”.

**Table 5:** Reasons for opting out of HIV Testing by eligible clients screened in City of Harare, April 2019

Variable	Frequency *n* = 70	(%)
Clients eligible for HIV testing		
Long waiting time before an HIV test	25	36
Health workers attitudes (Client Perception)	2	3
Fear of a positive result	15	21
Lack of privacy (Documentation of names)	5	7
Perceived low risk of contracting HIV	15	21
To consult partner	2	3
Reason not specified	6	9

**Table 6:** Reasons for failing to meet the intensified PITC target, Harare City, 2017–2018

Variable	Frequency *n* = 45	(%)
Health Workers		
High workload	21	47
Poor Patient flow	9	20
Shortage of staff	14	31
Shortage of test kits	1	2

## Discussion

We evaluated the intensified PITC intervention in 15 City of Harare clinics for the period October 2017 to September 2018. Several areas were noted to have gaps affecting proper implementation of the intensified PITC intervention. The inputs injected and processes used to implement the intervention were adequate to meet the set objectives. In this study adequacy was defined as an average of above (95%) achievement in terms of availability of inputs. However, some of the facilities did not have adequate testing space. The challenge was mainly experienced in the morning due to the high number of clients waiting to be tested after the eligibility screening process. Training of more health workers to beef up testing points in the morning and scaling up HIV self-testing may be necessary to increase uptake.

Unavailability of IEC material was a major bottleneck which may have negatively impacted on the intervention resulting in more clients opting out of HIV testing due to lack of information. The findings were consistent with the results of a study conducted in Ethiopia 2017 which showed that, the major reasons for not accepting the HIV test were poor access to correct understandable PITC information from health workers [12]. Hence, demand creation in the form of awareness campaigns and availing IEC materials in strategic positions is highly recommended.

During the period reviewed, a significant number of clients who were eligible for HIV testing at the 15 facilities were not tested for HIV. This is of great concern, considering the difficulties in convincing clients to come to health facilities for HIV testing. When they come to the facilities, the health system should ensure services are provided. Some of the health system factors identified to affect HIV testing in this study were too few testers and inadequate working space especially during peak hours. While some of the client related reasons were fear of a positive result and perceived low risk of contracting HIV. These important findings provide an opportunity for program managers to develop strategies to address identified testing gaps. The interventions will eventually contribute to the achievement of the UNAIDS first 90 which aims at identifying 90% of people living with HIV by 2020 [13].

Almost half of the health workers interviewed reported that high workload was the major reason for not meeting the target of testing all eligible clients. Despite the Human Resources for Health (HRH) support from the implementing partner, majority of the respondents indicated shortage of staff as the reason for not meeting their target. The challenge of workload was experienced mostly during the morning when the number of clients to be tested was high. The other contributory factor was that not all nurses were trained to test for HIV. The above findings were consistent with results of a study conducted in sub-Saharan Africa which was reviewing operational implementation of PITC program. Human resources and health systems management issues affected implementation of PITC [14]. Poor patient flow was also reported by a third of the respondents indicating the need for managers to have sound knowledge of the program to improve the system. Managers should ensure availability of adequate human and material resources to prevent interruption of services. A study conducted in South Africa in 2013 showed that strong leadership and implementation support by managers and good knowledge of the program by nurses aided in achieving the desired outcome [15].

Despite having been provided with information concerning the need to have an HIV test, a third of the eligible respondents who were individually screened using a screening tool still opted out. Respondents reported that the waiting time before being tested by the Primary Counsellor was too long especially in the morning. However, when clients wait too long before they are tested, they will opt out. Similarly, more than half of the respondents in a study conducted in Tanzania reported too many patients waiting for services as a reason for not testing. Another 46% in the same study reported inadequate space as the barriers to HIV testing [16]. We also found out that majority of the respondents did not agree to testing because of perceived low risk of contracting HIV and fear of a positive result. The self-risk perception to HIV infection was consistent with a study conducted in Ethiopian 2017 [12]. It is therefore necessary to intensify counselling so that clients are not afraid of an HIV test.

### Limitations

Our study was subjected to information bias because of the sensitive nature of some responses. Participants may not have provided correct information on reasons for opting out of HIV testing to protect their relationship with nurses. However, participants were assured of confidentiality and the bias was minimised. In addition, we opted for convenience sampling because of limited time which may have introduced selection bias and affected representativeness of the study population.

## Conclusion

The findings from our study shows that HIV testing coverage for eligible clients at the clinics was not optimal due to health system and client related reasons. The testing gap will negatively impact on the achievement of UNAIDS testing targets and eventually HIV epidemic control. Awareness campaigns through distribution of information education and communication materials on the importance of testing is desirable. Health facility systems such as annual refresher trainings, review of patient flow and re-allocation of staff during busy HIV testing time and use of HIV self-test kits for clients concerned with waiting time may help to improve HIV testing coverage.

## Supplementary Information

**Additional file 1 **: **Supplementary file 1.** Questionnaire with questions for health care workers and exit interview questions for clients.

## Data Availability

The data that support the findings of this study are available from the City of Harare Department of Health Services, but restrictions apply to the availability of these data. Data are, however, available from the authors upon request and with permission from City of Harare.

## Abbreviations

IPITC: Intensified Provider Initiated Testing & Counselling
PITC: Provider Initiated Testing & Counselling
AIDS: Acquired Immune Deficiency Syndrome
HIV: Human Immune Virus
PLWHIV: People Living with Human Immune Virus
ART: Antiretroviral therapy
ARV: Antiretroviral
OI: Opportunistic Infection
WHO: World Health Organisation?
UNAIDS: Joint United Nations Programme on HIV/AIDS
ZIMPHIA: Zimbabwe Population-Based HIV Impact Assessment
