# Supplementary material for: Evaluation of intensified provider initiated testing and counselling program in Harare City, 2017–2018
Source: BMC Public Health. 2021 Mar 2;21:431. doi: 10.1186/s12889-021-10485-6 (PMC7927239; doi:10.1186/s12889-021-10485-6)
Supplement: Supplementary file 1 — Additional file 1 : Supplementary file 1. Questionnaire with questions for health care workers and exit interview questions for clients. [file 12889_2021_10485_MOESM1_ESM.docx]

**1: Health Care Workers’ Questionnaire**

Questionnaire Number________ Date of interview _____________

**Demographic Characteristics**

1. Gender: Male_____/Female______
2. What is your current position title? (*tick the correct one*)

Sister in charge [ ]

OI Nurse [ ]

Primary Counsellor [ ]

1. What is the length of your service as worker in City of Harare? ________________________________

**Questions for process indicators**

1. Were you sensitised to implement the IPITC program during its inception in Harare? Yes__/No__. If yes was it:

formal __________________________

On the job ________________________

1. Did you receive any support and supervision visits from your supervisors in the last 12months? Yes_/No. If yes:

How many times? _______________________________

Were you given feedback? Yes_/No_

1. How often are Health Education talks conducted at your facility? *(tick the correct one)*

Daily [ ] Weekly [ ] Bi-weekly [ ] Monthly [ ] None [ ]

Other (specify) __________________

1. What type of health education sessions are held?

Group session [ ]

Individual session [ ]

Both group and individual [ ]

1. On average, how much time do you spend with a client when performing an HIV test?

About 5 minutes [ ]

6-10minutes [ ]

More than 10minutes [ ]

Depends on client need [ ]

Other (*specify*)___________________________

**Reasons for failing to meet the target.**

1. During implementation of IPITC, what are some of the main challenges that you experienced? (*tick correct response*)

Lack of adequate skills [ ]

Shortage of test kits [ ]

Shortage of staff [ ]

High workload [ ]

Unavailability of IEC Material [ ]

Inadequate working space [ ]

Poor patient flow system [ ]

Other (Specify) [ ]_______________________________

**2: Exit Interview Questionnaire for Clients**

Questionnaire number____________ Date of interview ____________

**Demographic Characteristics**

1. What is your age in completed years? ________________
2. What is your marital status?

Married [ ] Single [ ] Divorced [ ] Widowed [ ] Co-habiting [ ]

1. What is your highest level of education you attained?

Primary [ ] Secondary [ ] Tertiary [ ] Never been to school [ ]

1. What is your current employment status?

Formal [ ] Informal [ ] Not employed [ ]

1. On average how much money do you get as a family per month (Zimbabwe dollar)?

<$100 [ ] 100-500 [ ] >500 [ ]

1. Have you ever been tested for HIV? Yes_/No

If yes, when __________and what was the reason for testing**?**

**Reasons for Opting out of HIV testing.**

1. What are your reasons for opting out of HIV testing ?

- Long waiting time [ ]
- Perceived low risk of contracting HIV [ ]
- Health care workers attitudes [ ]
- Fear of a positive result [ ]
- Lack of privacy [ ]
- No Reason [ ]
- Others (Specify)_____________________________
